# Sepsis biomarkers: a review

**DOI:** 10.1186/cc8872

**Published:** 2010-02-09

**Authors:** Charalampos Pierrakos, Jean-Louis Vincent

**Affiliations:** 1 Department of Intensive Care, Erasme Hospital, Université Libre de Bruxelles, route de Lennik 808, 1070 Brussels, Belgium

## Abstract

**Introduction:**

Biomarkers can be useful for identifying or ruling out sepsis, identifying patients who may benefit from specific therapies or assessing the response to therapy.

**Methods:**

We used an electronic search of the PubMed database using the key words "sepsis" and "biomarker" to identify clinical and experimental studies which evaluated a biomarker in sepsis.

**Results:**

The search retrieved 3370 references covering 178 different biomarkers.

**Conclusions:**

Many biomarkers have been evaluated for use in sepsis. Most of the biomarkers had been tested clinically, primarily as prognostic markers in sepsis; relatively few have been used for diagnosis. None has sufficient specificity or sensitivity to be routinely employed in clinical practice. PCT and CRP have been most widely used, but even these have limited ability to distinguish sepsis from other inflammatory conditions or to predict outcome.

## Introduction

Sepsis is a leading cause of death in critically ill patients despite the use of modern antibiotics and resuscitation therapies [1]. The septic response is an extremely complex chain of events involving inflammatory and anti-inflammatory processes, humoral and cellular reactions and circulatory abnormalities [2,3]. The diagnosis of sepsis and evaluation of its severity is complicated by the highly variable and non-specific nature of the signs and symptoms of sepsis [4]. However, the early diagnosis and stratification of the severity of sepsis is very important, increasing the possibility of starting timely and specific treatment [5,6].

Biomarkers can have an important place in this process because they can indicate the presence or absence or severity of sepsis [7,8], and can differentiate bacterial from viral and fungal infection, and systemic sepsis from local infection. Other potential uses of biomarkers include roles in prognostication, guiding antibiotic therapy, evaluating the response to therapy and recovery from sepsis, differentiating Gram-positive from Gram-negative microorganisms as the cause of sepsis, predicting sepsis complications and the development of organ dysfunction (heart, kidneys, liver or multiple organ dysfunction). However, the exact role of biomarkers in the management of septic patients remains undefined [9]. C-reactive protein (CRP) has been used for many years [10,11] but its specificity has been challenged [12]. Procalcitonin (PCT) has been proposed as a more specific [13] and better prognostic [14] marker than CRP, although its value has also been challenged [15]. It remains difficult to differentiate sepsis from other non-infectious causes of systemic inflammatory response syndrome [16], and there is a continuous search for better biomarkers of sepsis.

With this background in mind, we reviewed the literature on sepsis biomarkers that have been used in clinical or experimental studies to help better evaluate their utility.

## Materials and methods

The entire Medline database was searched in February 2009 using the key words 'sepsis' and 'biomarker'. All studies, both clinical and experimental, which evaluated a biomarker were included. For each identified biomarker, the Medline database was searched again using the biomarker name and the key word 'biomarker'.

## Results

A total of 3370 studies that assessed a biomarker in sepsis were retrieved; 178 different biomarkers were evaluated in the 3370 studies. The retrieved biomarkers and the major findings from key studies using these biomarkers are listed in Tables 1, 2, 3, 4, 5, 6, 7, 8 and 9. Of the 178 biomarkers, 18 had been evaluated in experimental studies only, 58 in both experimental and clinical studies, and 101 in clinical studies only. Thirty-four biomarkers were identified that have been assessed for use specifically in the diagnosis of sepsis (Table 10); of these just five reported sensitivity and specificity values greater than 90%.

**Table 1:** Cytokine/chemokine biomarkers identified in the literature search (with some selected references)

Sepsis marker	Evaluated in experimental studies	Evaluated in clinical studies	Evaluated as a prognostic factor	Comment
GRO-alpha [49,50]	√	C (m)	√	Higher in septic shock than in sepsis
High mobility group-box 1 protein (HMGB-1) [51,52]	√	C	√	No difference between survivors and non-survivors at 28 days
IL-1 receptor antagonist [53-55]	√	A	√	Correlation with SOFA score
IL-1β [56,57]	√	A		Increased in septic compared with non-septic individuals
IL-2 [58]		B	√	Increased in parallel with disease severity
IL-4 [59]		C (s)	√	Increased levels associated with development of sepsis
IL-6 [48,60]	√	B	√*	Distinguished between survivors and non-survivors at 28 days
IL-8 [61,62]		B	√***	Prediction of MOF, DIC
IL-10 [63-65]	√	B	√**	Higher in septic shock than sepsis, distinguished between survivors and non-survivors at 28 days
IL-12 [66,67]	√	C	√	Predictive of lethal outcome from postoperative sepsis
IL-13 [68,69]	√	B	√	Higher in septic shock than sepsis
IL-18 [37,70]	√	B(s)	√	Distinguished between survivors and non-survivors at 28 days
Macrophage inflammatory protein (MIP)-1 and- 2 [71,72]	√	A	√	Increased in sepsis compared with healthy controls
Macrophage migration inhibitory factor (MIF) [42,73]	√	A	√**	Distinguished between survivors and non-survivors at 28 days
Monocyte chemotactic protein (MCP)-1 and 2 [42,74]	√	B	√*	Distinguished between survivors and non-survivors at 28 days
Osteopontin [75]		B		Increased in sepsis compared with healthy controls
RANTES [76,77]	√	B		Increased in sepsis compared with healthy controls
TNF [78,79]	√	C	√	Distinguished between survivors and non-survivors at 28 days in patients with septic shock

*sensitivity and specificity of less than 90%; **sensitivity of more than 90% but specificity of less than 90%; ***sensitivity and specificity more than 90%; A, Clinical study with less than 20 patients; B, Clinical study with 20 to 50 patients; C, Clinical study with more than 50 patients; (s), surgical patients only; (m), medical patients only.

DIC: disseminated intravascular coagulopathy; MOF: multiple organ failure; SOFA: sequential organ failure assessment.

**Table 2:** Cell marker biomarkers identified in the literature search (with some selected references)

Sepsis Marker	Evaluated in experimental studies	Evaluated in clinical studies	Evaluated as a prognostic factor	Comment
CD10 [80,81]	√	A		Decreased in septic shock compared with healthy controls
CD11b [82,83]	√	B(s)	√	Correlation with SOFA score
CD11c [84]		A		Decreased in septic shock compared with healthy controls
CD14 (cellular and soluble) [85]		C	√	Distinguished between survivors and non-survivors at 28 days
CD18 [86]	√			
CD25 (cellular and soluble) [87]		A		Distinguished between survivors and non-survivors at 28 days
CD28 (soluble) [88]		B	√	Distinguished between survivors and non-survivors at 28 days
CD40 (cellular and soluble) [89]		B	√	Distinguished between survivors and non-survivors at 28 days
CD48 [90]		B		Increased in sepsis compared with healthy controls
CD64 [91]		B	√	Correlation with APACHE II and SOFA scores
CD69 [92]		A		Increased in sepsis compared with healthy controls
CD80 [88]		B	√	Predicted development of septic shock
CD163 (soluble) [93]		C	√	Distinguished between survivors and non-survivors at 28 days
mHLA-DR (soluble) [94]		C	√*	Distinguished between survivors and non-survivors at 28 days in patients with septic shock

*sensitivity and specificity of less than 90%; A, Clinical study with less than 20 patients; B, Clinical study with 20 to 50 patients; C, Clinical study with more than 50 patients; (s), surgical patients only.

APACHE: acute physiology and chronic health evaluation; SOFA: sequential organ failure assessment.

**Table 3:** Receptor biomarkers identified in the literature search (with some selected references)

Sepsis marker	Evaluated in experimental studies	Evaluated in clinical studies	Evaluated as a prognostic factor	Comment
CC chemokine receptor (CCR) 2 [95]	√			
CCR 3 [96]		C	√	Distinguished between survivors and non-survivors at 28 days
C5L2 [97]	√	B	√	Predicted development of MOF
CRTh2 [98]		C	√	Distinguished between survivors and non-survivors at 28 days
Fas receptor (soluble) [99]		B(m)	√	Predicted development of MOF
Fc-gamma RIII [100]		A	√	Increased in sepsis compared with healthy controls, correlated with APACHE II score
FLT-1 (soluble) [101,102]	√	C	√	Correlated with APACHE II score
GP130 [103]		A		Increased in sepsis compared with healthy controls
IL-2 receptor (soluble) [104]		C	√	Predicted development of septic shock
Group II phospholipase A2 (PLA2-II) (soluble) [105,106]	√	B		Distinguished between survivors and non-survivors at 28 days
RAGE (soluble) [107]		B	√*	Distinguished between survivors and non-survivors at 28 days
ST2 (soluble, IL-1 receptor) [108]		A(s)	√	Increased in sepsis compared with healthy controls
Toll-like receptor (TLR) 2 and 4 [109]	√	B	√	Increased in septic compared with non-septic critically ill patients
Transient receptor potential vanilloid (TRPV)1 [110]	√			
TREM-1 (soluble) [111,112]	√	C	√	Distinguished between survivors and non-survivors at 28 days
TNF-receptor (soluble) [113]		B		Predicted development of MOF
Urokinase type plasminogen activator receptor (uPAR) (soluble) [114]		C(m)	√	Distinguished between survivors and non-survivors at 28 days

*sensitivity and specificity of less than 90%; A, Clinical study with less than 20 patients; B, Clinical study with 20 to 50 patients; C, Clinical study with more than 50 patients; (s), surgical patients only; (m), medical patients only.

APACHE: acute physiology and chronic health evaluation; MOF: multiple organ failure; TREM: triggering receptor expressed on myeloid cells; RAGE: receptor for advanced glycation end-products.

**Table 4:** Coagulation biomarkers identified in the literature search (with some selected references)

Sepsis marker	Evaluated in experimental studies	Evaluated in clinical studies	Evaluated as a prognostic factor	Comment
Antithrombin [115]	√	B	√**	Distinguished between survivors and non-survivors at 28 days
Activated partial thromboplastin time (aPTT) [35]		C	√	Correlated with MOF score in patients with sepsis and DIC, high negative predictive value
D-dimers, TAT, F1.2, PT [116]		C	√	Distinguished between survivors and non-survivors at 28 days, correlated with APACHE II score
Fibrin [36]		C		Increased in patients with Gram-negative bacteremia
PF-4 [117]		A	√	Predicted response to therapy
Plasminogen activator inhibitor (PAI)-1 [118,119]		B	√	Distinguished between survivors and non-survivors at 28 days, predicted development of MOF
Protein C and S [120,121]	√	C	√*	Distinguished between survivors and non-survivors at 28 days
Thrombomodulin [122,123]	√	C	√	Predicted development of MOF, DIC, and response to therapy

*sensitivity and specificity of less than 90%; **sensitivity of more than 90% but specificity of less than 90%; A, Clinical study with less than 20 patients; B, Clinical study with 20 to 50 patients; C, Clinical study with more than 50 patients.

APCHE: acute physiology and chronic health evaluation; DIC: disseminated intravascular coagulopathy; MOF: multiple organ failure; PT: prothrombin time; PF: platelet factor; TAT: thrombin-antithrombin complex.

**Table 5:** Biomarkers related to vascular endothelial damage identified in the literature search (with some selected references)

Sepsis marker	Evaluated in experimental studies	Evaluated in clinical studies	Evaluated as a prognostic factor	Comment
ADAMTS-13 [124,125]	√	B	√	Decreased in septic patients with DIC compared with no DIC
Angiopoietin (1 and 2) [126]		B	√	Distinguished between survivors and non-survivors at 28 days
Endocan [127,128]	√	B	√	Predicted development of septic shock
Endothelial leukocyte adhesion molecule (ELAM)-1 (cellular and soluble) [129,130]	√	B(s)	√*	Distinguished between survivors and non-survivors at 28 days
Endothelial progenitor cells (cEPC) [131]		B	√	Distinguished between survivors and non-survivors at 28 days
Intracellular adhesion molecule (ICAM)-1 (soluble) [38]	√	B(m)	√	
Laminin [132]		A		Increased in sepsis compared with non-infected controls
Neopterin [133,134]	√	C	√*	Distinguished between survivors and non-survivors at 28 days
Platelet-derived growth factor (PDGF)-BB [135]		B	√	Distinguished between survivors and non-survivors at 28 days in patients with severe sepsis
E-Selectin (cellular and soluble) [123,136]	√	C	√	Predicted development of MOF, correlated with SAPS score
L-Selectin (soluble) [137]		C	√*	Distinguished between survivors and non-survivors at 28 days
P-Selectin [138]	√			
Vascular cell adhesion molecule (VCAM)-1 [139,140]	√	C		Predicted development of MOF
Vascular endothelial growth factor (VEGF) [141,142]	√	A	√	Distinguished between survivors and non-survivors at 28 days, predicted development of MOF
von Willebrand factor and antigen [143,144]		B(m)	√	Distinguished between survivors and non-survivors at 28 days, predicted development of acute lung injury

*sensitivity and specificity of less than 90%; A, Clinical study with less than 20 patients; B, Clinical study with 20 to 50 patients; C, Clinical study with more than 50 patients; (s), surgical patients only; (m), medical patients only.

DIC: disseminated intravascular coagulopathy; MOF: meultiple organ failure; SAPS: simplified acute physiology score.

**Table 6:** Biomarkers related to vaosdilation identified in the literature search (with some selected references)

Sepsis marker	Evaluated in experimental studies	Evaluated in clinical studies	Evaluated as a prognostic factor	Comment
Adrenomedullin and pro-adrenomedullin [145,146]		B	√*	Predicted development of septic shock
Anandamide [147]	√	A		Increased in sepsis compared with healthy controls
Angiotensin converting enzyme (ACE) (activity and serum) [148,149]	√	B		Increased in sepsis compared with healthy controls
2-arachidonoylglycerol [150]		A		Increased in sepsis compared with healthy controls
Copeptin [151]		C(m)	√*	Distinguished between survivors and non-survivors at 28 days, correlated with APACHE II score
C-type natriuretic peptide (CNP) [152]		A		Increased in patients with septic shock compared with healthy controls
Cycling nucleotides [153,154]	√	A(m)	√	Distinguished between survivors and non-survivors at 28 days
Elastin [155]		B		Decreased in sepsis compared with healthy controls
cGRP [156,157]	√	C(s)	√	Distinguished between survivors and non-survivors at 28 days, correlated with APACHE II score
47 kD HK [158]		B(m)		Correlated with severity of sepsis
Neuropeptide Y [159,160]	√	A		Increased in sepsis compared with healthy controls
Nitric oxide (NO), nitrate, nitrite [161,162]	√	B	√	Predicted development of septic shock
Substance P [156,163]	√	C(s)	√	Distinguished between survivors and non-survivors at 28 days (predictive only in the late phase of sepsis, 2 days before death)
Tetrahydrobiopterin [164,165]		A		Increased in sepsis compared with non-septic critically ill patients
Vasoactive intestinal peptide (VIP) [166,167]	√	A		Increased in tissue samples from patients with peritonitis compared with no peritonitis

*sensitivity and specificity of less than 90%; A, Clinical study with less than 20 patients; B, Clinical study with 20 to 50 patients; C, Clinical study with more than 50 patients; (s), surgical patients only; (m), medical patients only.

APACHE: acute physiology and chornic health evaluation; cGRP: calcitonin gene-related peptide; HK: high-molecular weight kininogen.

**Table 7:** Biomarkers of organ dysfunction identified in the literature search (with some selected references)

Sepsis marker	Evaluated in experimental studies	Evaluated in clinical studies	Evaluated as a prognostic factor	Comment
Atrial natriuretic peptide (ANP) [168,169]		C	√*	Distinguished between survivors and non-survivors at 28 days
Brain natriuretic peptide (BNP) [170-172]		B	√**	Distinguished between survivors and non-survivors at 28 days, correlated to APACHE II score
Carbomyl phosphate synthase (CPS)-1 [173]	√			
Endothelin-1 and pro-endothelin-1 [174-177]	√	B	√	Distinguished between survivors and non-survivors at 28 days, correlated with SOFA score
Filterable cardiodepressant substance (FCS) [178]	√			
Gc-globulin [179]		C(s)		Predicted development of MOF
Glial fibrillary acidic protein (GFAP) [180]		B	√	Increased in septic shock compared with healthy controls
alpha glutathione S-transferase (GST) [181]	√			
Hepatocyte growth factor (HGF) (cellular and soluble) [182,183]	√	C(m)		Predicted response to therapy
MEGX test [184,185]	√	A	√	Correlated with SAPS II score
Myocardial angiotensin II [186]	√			
NSE [187]		B	√	Correlated with SOFA scores
Pancreatitis-associated protein-I [188]	√			
Pre B cell colony-enhancing factor (PBEF) [189]		A		Increased in sepsis compared with healthy controls
Protein S-100b [187,190]	√	B	√	Distinguished between survivors and non-survivors at 28 days, correlated with SOFA score
Surfactant protein (A, B, C, D) [191,192]	√	A		Increased in sepsis compared with healthy controls
Troponin [193]		B	√	Distinguished between survivors and non-survivors at 28 days, correlated with APACHE II score

*sensitivity and specificity of less than 90%; **sensitivity of more than 90% but specificity of less than 90%; A, Clinical study with less than 20 patients; B, Clinical study with 20 to 50 patients; C, Clinical study with more than 50 patients; (s), surgical patients only; (m), medical patients only.

APACHE: acute physiology and chronic health evaluation; MEGX: monoethylglycinexylidide; MOF: multiple organ failure; NSE: neuron-specific enolase; SAPS: simplified acute physiology score; SOFA: sequential organ failure assessment.

**Table 8:** Acute phase protein biomarkers identified in the literature search (with some selected references)

Sepsis Marker	Evaluated in experimental studies	Evaluated in clinical studies	Evaluated as a prognostic factor	Comment
Serum amyloid A (SAA) [194,195]	√	B(s)	√	Correlated with CRP in patients with septic shock
Ceruloplasmin [196,197]		A	√	Predicted liver dysfunction in patients with sepsis
C-reactive protein (CRP) [11,198,199]		C	√*	Predicted response to therapy
Ferritin [200]		B(m)	√	Distinguished between survivors and non-survivors at 28 days, correlated with SOFA score
Alpha1-acid glycoprotein [201,202]	√	B	√	Distinguished between survivors and non-survivors at 28 days, correlated with SOFA score
Hepcidin [203]		B		Incraesed in sepsis compared with healthy controls and patients with chronic renal failure
Lipopolysaccharide binding protein (LBP) [39,204]	√	C(s)	√	Higher in sepsis compared with no sepsis, no prognostic value
Procalcitonin [21,134,205]	√	C	√*	Increased in infected compared with non-infected patients
Pentraxin 3 [206,207]		C	√	Distinguished between survivors and non-survivors at 28 days, correlated with APACHE II score

*sensitivity and specificity of less than 90%; A, Clinical study with less than 20 patients; B, Clinical study with 20 to 50 patients; C, Clinical study with more than 50 patients; (s), surgical patients only; (m), medical patients only.

APACHE: acute physiology and chronic health evaluation; SOFA: sequential organ failure assessment.

**Table 9:** Other biomarkers identified in the literature search (with some selected references)

Sepsis marker	Evaluated in experimental studies	Evaluated in clinical studies	Evaluated as a prognostic factor	Comment
Alpha2 macroglobulin [196,208]	√			
Albumin [209]	√			
Anti-endotoxin core antibodies (EndoCab) [210]		A	√	Distinguished between survivors and non-survivors at 28 days
Apolipoprotein CI [211-213]		C	√	Distinguished between survivors and non-survivors at 28 days
Bcl-2 [214]		A	√	Distinguished between survivors and non-survivors at 28 days
Beta-thromboglobulin [215]		B	√	Predicted response to therapy
Caspase-1 [216]		A		Increased in septic shock compared with healthy controls
Ceramide [217]		B	√**	Predicted development of MOF
Cholesterol [218]		C	√	Distinguished between survivors and non-survivors at 28 days in patients with severe sepsis
Complement (C3, C4, C5a levels) [219,220]		B(m)	√	Distinguished between survivors and non-survivors at 28 days
Terminal complement complex [221]	√			
Dendritic cell [222,223]	√	B	√	Distinguished between survivors and non-survivors at 28 days, correlated with SOFA score
Dipeptidylpeptidase [224]		B		Decreased in sepsis compared with healthy controls
Diiodotyrosine (DIT) [225]		C	√	Increased in sepsis compared with non-septic critically ill
Eicosanoid [226,227]	√	A(s)	√	Correlated with SAPS score, predicted response to therapy
Elastase [228,229]	√	C(s)	√	Predicted response to therapy in patients with joint infections
Elastase-a1-antitrypsin complex [230,231]		C	√	Predicted response to therapy
Erythropoietin [232]		A	√	Distinguished between survivors and non-survivors at 28 days in patients with septic shock, correlated with lactate levels
F2 isoprostanes [233]		B(m)	√	Increased in infected diabetic patients compared with non-infected diabetics
Fatty acid amide hydrolase [234]		A	√	Decreased in sepsis compared with healthy controls
Free DNA [235]		B	√*	Distinguished between survivors and non-survivors at 28 days
G-CSF and GM-CSF [236,237]		B	√**	Distinguished between survivors and non-survivors at 28 days
Gelsolin [238]		B(s)	√	Distinguished between survivors and non-survivors at 28 days
Ghrelin [239,240]	√			
Growth arrest specific protein (Gas) 6 [241]		B	√	Correlated with APACHE II score in patients with severe sepsis
Heat shock protein (HSP)70, 72, 73, 90 and 32 [242-245]	√	C(s)		Increased in sepsis compared with healthy controls
HDL cholesterol		C	√**	Distinguished between survivors and non-survivors at 28 days, predicted polonged ICU length of stay
HLA-G5 protein *(soluble) *[246]		C(m)	√*	Distinguished between survivors and non-survivors at 28 days in patients with septic shock
H_2_S [247]	√			
Hyaluronan [248,249]	√	B		Distinguished between survivors and non-survivors at 28 days in patients with septic shock
Hydrolytic IgG antibodies [250]		B	√	Distinguished between survivors and non-survivors at 28 days, correlation with SAPS II score
Inter-alpha inhibitor proteins (IalphaIp) [251]		C	√	Predicted development of MOF
Intracellular nitric oxide in leukocyte [252]		B	√	Negatively correlated with SOFA score
IP-10 [30]		C		Increased in sepsis compared with healthy controls
Lactate [253,254]		C	√	Distinguished between survivors and non-survivors at 28 days, predicted response to therapy
Lactoferrin [255,256]	√	C(s)		Predicted response to therapy
Leptin [240,257]	√	B	√	No prognostic value, higher in septic than in non-septic ICU patients
Serum lysozyme (enzyme activity) [258]		B(s)		Increased in sepsis compared with healthy controls
Matrix-metalloproteinase (MMP)-9 [259]		B		Increased in severe sepsis compared with healthy controls
Microparticles (cell derived) [252]		B	√	Distinguished between survivors and non-survivors at 28 days, correlation with SOFA score
Neurotensin [260]	√			
Nitrate excretion (urinary and expired) [261]	√			
Nociceptin/orphanin FQ (N/OFQ) [262]		A	√	Distinguished between survivors and non-survivors at 28 days
NF-κB (activity and expression) [263]		B	√**	Distinguished between survivors and non-survivors at 28 days in patients with severe sepsis, correlation with APACHE II score
Nucleosomes [264]		C		Distinguished between survivors and non-survivors at 28 days
Peptidoglycan [265]		B(s)	√	Increased in sepsis compared with healthy controls
PlGF [266]	√			
Plasma amino acids [267-269]		A	√	Distinguished between survivors and non-survivors at 28 days, predicted development of MOF
Plasma fibronectin [270]		B	√	Increased in sepsis compared with healthy controls
Plasmin alpha2-antiplasmin complex [271]		C		Predicted development of MOF
Renin [272]		B	√	Correlation with lactate levels in patients with septic shock
Resistin [273]		C	√	Correlation with APACHE II score in patients with severe sepsis
Selenium [274]		C	√	Correlation with APACHE II in patients with severe sepsis
Selenoprotein P [275]		B		Decraesed in sepsis compared with healthy controls
Serum bicarbonate [276]		A(m)	√	Predicted development of septic shock in neutropenic patients
Sphingomyelinase (enzyme activity) [277]		A		Distinguished between survivors and non-survivors at 28 days in patients with severe sepsis
Sulfite [278]	√	B(m)	√	Predicted response to therapy
Transforming growth factor (TGF)-β1 [279,280]	√	A(m)		Distinguished between survivors and non-survivors at 28 days
TIMP-1 and 2 [259]		B	√*	Distinguished between survivors and non-survivors at 28 days
TIMP-3 [281]	√			
Uric acid [282]		C(s)	√	Decreased in postoperative patients with sepsis compared with those with no sepsis
Urinary 8-OhdG [283]		C	√	Distinguished between survivors and non-survivors at 28 days
Urinary bilirubin oxidative metabolites (BOMs) [284]		A	√	Correlation with APACHE II score
Annexin V binding [285]	√	B(s)		Increased in sepsis compared with healthy controls
Xanthine oxidase (activity) [286]		C	√	Distinguished between survivors and non-survivors at 28 days

*sensitivity and specificity of less than 90%; **sensitivity of more than 90% but specificity of less than 90%; A, Clinical study with less than 20 patients; B, Clinical study with 20 to 50 patients; C, Clinical study with more than 50 patients; (s), surgical patients only; (m), medical patients only.

APACHE: acute physiology and chronic health evalution; G-CSF: granulocyte colony-stimulating factor; GM-CSF: granulocyte-macrophage colony stimulating factor; MOF: multiple organ failure; NF-κB: nuclear factor kappa B; PlGF: placental growth factor; SAPS: simplified acute physiology score; SOFA: sequential organ failure assessment; TIMP: tissue inhibitor of metalloproteinase.

**Table 10:** Biomarkers that have been assessed for use in the diagnosis of sepsis

	Sepsis biomarker	Clinical study	Type of measurement	Outcome
1	aPTT** [35]	**C**	**c**	High negative predictive value
2	CD11b*** [33]	**B**	**s**	Higher values in neonates with sepsis than in those with possible infection
3	**CD25 **[87]	**A**	**s**	Distinguished between sepsis and SIRS
4	CD64*** [32,287]	**C**	**s**	Low sensitivity and specificity to distinguish between viral and bacterial infections
5	**Complement (C3, C4, C5a) **[219]	**B**	**s**	Distinguished between sepsis and SIRS
6	**EA complex **[230]	**C**	**s**	Diagnosis of sepsis, increased earlier than CRP
7	**ELAM-1 (cellular and soluble) **[129]	**C(s)**	**c**	Increased in trauma patients with sepsis compared with no sepsis
8	Endocan [127]	**B**	**s**	Distinguished between sepsis and SIRS
9	**E-Selectin (cellular and soluble) **[136]	**B**	**s**	Distinguished between sepsis and SIRS
10	**Fibrin degradation products **[36]	**B**	**s**	High negative predictive value
11	**Gas6 **[241]	**B**	**s**	Higher values in patients with severe sepsis compared with patients with organ failure but no sepsis
12	**G-CSF **[237]	**C**	**s**	Distinguished between sepsis and SIRS
13	**Gelsolin **[238]	**B(s)**	**c**	Higher in septic patients compared with patients without sepsis
14	**IL-1 receptor antagonist **[55]	**C**	**s**	Early diagnosis of sepsis before symptoms in newborns
15	IL-8* [61]	**C**	**s**	Higher in septic neutropenic patients compared with febril neutropenic patients without sepsis
16	**IL-10 **[65]	**A**	**s**	Higher in septic shock compared with cardiogenic shock
17	IL-12*** [29]	**C**	**s**	Diagnosis of sepsis in pediatric patients
18	**IL-18 **[70]	**B(s)**	**s**	Distinguished between Gram-positive and Gram-negative sepsis. Higher in trauma patients with sepsis than in those without
19	IP-10*** [30]	**C**	**s**	Early diagnosis of sepsis in newborns
20	**Laminin **[38]	**A**	**s**	Distinguished between Candida sepsis and bacterial sepsis
21	**LBP **[204]	**C**	**s**	Distinguished between Gram-positive sepsis and Gram-negative
22	**MCP-1 **[61]	**C**	**s**	Distinguished between sepsis and SIRS in neutropenic pediatric patients
23	**NO, nitrate, nitrite **[161]	**B**	**s**	Higher in septic shock compared with cardiogenic shock
24	**Osteopontin **[75]	**B**	**s**	Distinguished between sepsis and SIRS
25	**PAI-1 **[118]	**B**	**s**	Higher in patients with sepsis and DIC compared with no-septic patients with DIC
26	**Pentraxin 3 **[207]	**C**	**s**	Distinguished between septic shock and SIRS
27	**Peptidoglycan **[262]	**B(s)**	**c**	Higher in postoperative patients with infection compared with no-infected postoperative patients
28	**pFN **[270]	**B**	**s**	Distinguished between sepsis and SIRS
29	PLA2-II (soluble)*** [31]	**B**	**s**	Distinguished between bacteremic and non-bacteremic infections
30	**Serum lysozyme (enzyme activity) **[258]	**B**	**s**	Distinguished between sepsis and organ rejection in transplanted patients
31	**ST2 (soluble) **[108]	**A**	**s**	Higher in septic patients compared with those with no sepsis
32	**Surfactant protein (A, B, C, D) **[192]	**B**	**s**	Early diagnosis of ARDS in septic patients
33	**TREM-1 (soluble) **[288,289]	**C**	**s**	Distinguished between sepsis and SIRS, diagnosed pneumonia
34	**Troponin **[193]	**B**	**s**	Diagnosis of myocardial dysfunction in septic patients

*sensitivity and specificity of less than 90%; **sensitivity of more than 90% but specificity of less than 90%; ***sensitivity and specificity more than 90%; A, Clinical study with less than 20 patients; B, Clinical study with 20 to 50 patients; C, Clinical study with more than 50 patients; (s), surgical patients only; (m), medical patients only; s, single value; c, values over time.

aPTT: activated partial thromboplastin time; ARDS: acute respiratory distress syndrome; CRP: C-reactive protein; DIC: disseminated intravascular coagulopathy; EA: elastase alpha 1-proteinase inhibitor; ELAM: endothelial leukocyte adhesion molecule; G-CSF: granulocyte colony-stimulating factor; IP: interferon-induced protein; LBP: lipopolysaccharide-binding protein; MCP: monocyte chemotactic protein; NO: nitric oxide; PAI: plasminogen activator inhibitor; pFN: plasma fibronectin; PLA2: phospholipase A2; SIRS: systemic inflammatory response syndrome; TREM: triggering receptor expressed on myeloid cells.

## Discussion

A multitude of biomarkers has been proposed in the field of sepsis, many more than in other disease processes; for example, a study of patients with myocardial infarction revealed 14 biomarkers suitable for diagnosis and determination of prognosis [17] and in patients with Alzheimer's disease, just 8 biomarkers were identified [18]. This large difference in the numbers of biomarkers for sepsis is likely to be related to the very complex pathophysiology of sepsis, which involves many mediators of inflammation [19], but also other pathophysiological mechanisms. Coagulation, complement, contact system activation, inflammation, and apoptosis are all involved in the sepsis process, and separate markers for each (part of each) system have been proposed (Tables 1 to 9). Additionally, the systemic nature of sepsis and the large numbers of cell types, tissues and organs involved expand the number of potential biomarker candidates, compared with disease processes that involve individual organs or are more localized.

It is interesting to note that most of the biomarkers we identified have been tested clinically and not experimentally. This is likely to be in part related to difficulties creating an experimental model that accurately reflects all aspects of human sepsis, problems with species differences, and problems in determining end-points in animal studies. Additionally, as the sepsis response varies with time, the exact time period during which any specific biomarker may be useful varies, and this is difficult to assess reliably in experimental models. Moreover, as there is no 'gold standard' for the diagnosis of sepsis, the effectiveness of a biomarker needs to be compared with current methods used to diagnose and monitor sepsis in everyday clinical practice, i.e., by the combination of clinical signs and available laboratory variables [20]; experimental models cannot be used for this purpose.

Our study revealed that there are many more potential biomarkers for sepsis than are currently used in clinical studies. Some of these markers may require considerable time, effort and costs to measure. Some are already routinely used for other purposes and easily obtained, such as coagulation tests or cholesterol concentrations. In many cases, the reliability and validity of the proposed biomarker have not been tested properly [8]. Of the many proposed markers for sepsis, acute phase proteins have perhaps been most widely assessed. PCT has been used particularly extensively in recent years. The specificity and sensitivity of PCT for the diagnosis of sepsis is relatively low (typically below 90%), regardless of the cut-off value [21,22]. Raised PCT levels have also been reported in other conditions associated with inflammatory response, such as trauma [23], major surgery [24] and cardiac surgery [25]. Although CRP is often reported as inferior compared with PCT in terms of sepsis diagnosis, it is frequently used in clinical practice because of its greater availability. Elevated concentrations of serum CRP are correlated with an increased risk of organ failure and death [26], and the study of its time course may be helpful to evaluate the response to therapy in septic patients [11].

Another group of compounds that has been widely assessed as potential biomarkers are the cytokines. These are important mediators in the pathophysiology of sepsis, and most are produced fairly rapidly after sepsis onset. In a clinical study, levels of TNF and IL-10 were increased within the first 24 hours after admission of the patient [27]. However, blood cytokine concentrations are rather erratic and their time course is not clearly in concert with the course of sepsis [27,28], making interpretation difficult.

The diagnosis of sepsis is a challenge. Clinical and standard laboratory tests are not very helpful because most critically ill patients develop some degree of inflammatory response, whether or not they have sepsis. Even microbiological assessment is unreliable because many culture samples do not yield microorganisms in these patients. However, biomarkers have also not been shown to be a great asset in the diagnosis of sepsis. Indeed, relatively few biomarkers have been evaluated as diagnostic markers (Table 10). Our search retrieved only 10 biomarkers that have been assessed for their ability to distinguish septic patients from non-septic patients with systemic immune response syndrome. However, none of these biomarkers has been tested for both sensitivity and specificity, and there is therefore no biomarker clearly identified as being able to differentiate sepsis syndrome from an inflammatory response due to other causes.

Early diagnosis of sepsis is also an important issue as early institution of appropriate therapy, including antibiotics, is associated with improved outcomes. We identified 16 factors that have been evaluated specifically for the early diagnosis of sepsis; five of these had reported sensitivity and specificity of more than 90%. IL-12 was measured in newborns at the time when sepsis was first suspected clinically and was higher in patients with sepsis than in those without [29]. Interferon-induced protein 10 (IP-10) was higher in neonates with sepsis and necrotizing enterocolitis than in neonates who had only necrotizing enterocolitis [30]. These two biomarkers have not been evaluated for this purpose in adults. Group II phospholipase 2 (PLA2-II) was reported to have high sensitivity and specificity for the diagnosis of bacteremia in critically ill adult patients within 24 hours after admission [31]. CD64 had high sensitivity and specificity for the early diagnosis of sepsis in adults, but could not reliably distinguish viral from bacterial infections, or local infection from systemic sepsis [32]. Neutrophil CD11b could distinguish septic pediatric patients from those with possible infection with good sensitivity and specificity [33]. The sensitivity and specificity of the other 11 biomarkers used to diagnose early sepsis were not reported or were less than 90%.

Biomarkers can be more useful to rule out sepsis than to rule it in. We identified three biomarkers with high negative predictive value to rule out sepsis: PCT (99% at a cut-off value of 0.2 ng/ml) [34]; activated partial thromboplastin time (aPTT) waveform (96%) [35]; and fibrin degradation products (100% for Gram-negative sepsis by ELISA assay) [36]. It is important to emphasize that culture-positive sepsis was generally used as the gold standard in all these studies, although cultures may remain negative in many patients with sepsis.

The majority of the biomarkers that we identified in our search were assessed for their ability to differentiate patients likely to survive from those likely to die. Indeed, any biomarker is expected to have some prognostic value and sepsis biomarkers are no exception; however, this is not an absolute rule because some sepsis biomarkers failed to have prognostic value [37-39]. Moreover, sensitivity and specificity were tested in only some of the proposed prognostic markers, and none had sufficient (more than 90%) sensitivity and specificity to predict which patients were at greater risk of dying due to sepsis. Other biomarkers were assessed for their ability to predict the development of multiple organ failure and to evaluate response to therapy. It is known that the extent of infection and the severity of organ failure has a significant impact on the prognosis of patients with sepsis. Additionally, the response to therapy varies among patients. Recently, the PIRO model has been proposed as a way of stratifying septic patients according to their Predisposing condition, the severity of Infection, the Response to therapy and the degree of Organ dysfunction [20]. In the future, sepsis biomarkers may contribute to this model of classification rather than just being used as prognostic markers.

No biomarker has, therefore, established itself sufficiently to be of great help to clinicians in everyday clinical practice. As each biomarker has limited sensitivity and specificity, it may be interesting to combine several biomarkers [40,41]; however, this hypothesis requires further study. A clinical study showed that the combination of aPTT waveform with PCT increased the specificity of the aPTT waveform in the diagnosis of sepsis [35]. Studies using panels of sepsis biomarkers have also provided encouraging results [42-44]. The cost-effectiveness of all these methods must also be evaluated.

In this study, we tried to categorize the sepsis biomarkers according to their pathophysiological role in sepsis. A useful sepsis marker must not only help to identify or rule out sepsis, but it should also be able to be used to guide therapy. It has been shown that using PCT levels to guide therapy reduces antibiotic use and may be associated with improved outcomes [45,46]. The use of novel therapies that modify the pathophysiological process of sepsis may also be guided by biomarkers [47,48]. A study is underway to evaluate the value of protein C levels to guide the administration of activated protein C (clinicaltrials.gov identifier NCT00386425). In the future, sepsis biomarkers may help us administer these therapies to the right patient at the right time.

## Conclusions

Our literature review indicates that there are many biomarkers that can be used in sepsis, but none has sufficient specificity or sensitivity to be routinely employed in clinical practice. PCT and CRP have been most widely used, but even these have limited abilities to distinguish sepsis from other inflammatory conditions or to predict outcome. In view of the complexity of the sepsis response, it is unlikely that a single ideal biomarker will ever be found. A combination of several sepsis biomarkers may be more effective, but this requires further evaluation.

## Key messages

• More than 170 different biomarkers have been assessed for potential use in sepsis, more for prognosis than for diagnosis.

• None has sufficient specificity or sensitivity to be routinely employed in clinical practice.

• Combinations of several biomarkers may be more effective than single biomarkers, but this requires further evaluation.

## Abbreviations

aPTT: activated partial thromboplastin time; CRP: C-reactive protein; ELISA: enzyme-linked immunosorbent assay; IL: interleukin; IP-10: interferon-induced protein 10; PCT: procalcitonin; PLA2-II: group II phospholipase 2; TNF: tumor necrosis factor.

## Competing interests

The authors declare that they have no competing interests.

## Authors' contributions

CP and JLV conceived the study. CP conducted the literature search. CP and JLV wrote the manuscript.
